# Implementing polygenic risk-based breast cancer screening across European health systems: insights from the BRIGHT project

**DOI:** 10.3389/frhs.2026.1829295

**Published:** 2026-07-07

**Authors:** Mario del Vicario, Luciana Pons Muzzo, Kellie Harkin, Roger Marginet i Assens, Tiwatayo Lasebikan, Neeme Tõnisson, Peeter Padrik, Inna Feldman, Filipa Sampaio, Luis Costa, Gonçalo Nogueira-Costa, Madli Tamm, Krista Kruuv-Käo, Laetitia Paumard, Magda Rosenmöller

**Affiliations:** 1 IESE Business School; 2 Institute of Genomics, University of Tartu, Tartu, Estonia; 3 OÜ Antegenes, Tartu, Estonia; 4 Genetics and Personalized Medicine Clinic, Tartu University Hospital, Tartu, Estonia; 5 Hematology and Oncology Clinic, Tartu University Hospital, Tartu, Estonia; 6 Region Uppsala, Uppsala, Sweden; 7 Uppsala Health Economics, Department of Public Health and Caring Sciences, Uppsala University, Uppsala, Sweden; 8 Department of Oncology, Unidade Local de Saúde Santa Maria, Clinical Research Center – Lisbon Medical Academic Center, Lisbon, Portugal

**Keywords:** breast cancer screening, business models, digital health infrastructure, genomic medicine, health technology assessment, implementation science, personalized prevention, polygenic risk score (PRS)

## Abstract

**Introduction:**

Polygenic risk scores (PRS) offer a novel means of stratifying breast cancer risk, with the potential to personalize screening protocols and measures, and thus improve early detection. However, their implementation in real-world settings remains poorly understood. This study, as part of the BRIGHT project, investigates how PRS-based breast cancer screening is being piloted, adapted, and resisted across five European countries, Estonia, Portugal, Spain, Sweden, and France, drawing on a combination of implementation science and business model theory.

**Methods:**

We employed a multiple-case study approach, conducting 94 semi-structured interviews with stakeholders across healthcare, policy, industry, and civil society. Analysis was guided by the Value-Information-Process (VIP) framework and complemented by selected constructs from the Consolidated Framework for Implementation Research (CFIR). Data were coded thematically and triangulated with national policy documents, pilot evaluations, and stakeholder workshops.

**Results:**

Implementation trajectories varied significantly across contexts. Estonia demonstrated advanced policy-driven integration enabled by digital infrastructure and centralized governance. Sweden and Spain followed pilot-to-policy models, with strong regional autonomy but slower national uptake. Portugal exhibited market-led adoption driven by private sector innovation, while France pursued a trial-first logic centered on MyPeBS. Common barriers included limited clinical guidelines, low genetic literacy among frontline providers, fragmented IT systems, and slow and unclear Health Technology Assessment (HTA) cycles. Key facilitators included strong institutional alignment, biobank-linked infrastructures, and public trust in preventive care.

**Discussion:**

Successful PRS implementation requires more than scientific validation; it depends on systemic readiness across value narratives, information systems, and governance processes. PRS-based screening thus serves as a test case for the broader challenge of integrating personalized genomics into public health. Findings offer strategic guidance for policymakers, clinicians, and researchers designing future-ready prevention pathways.

## 1 Introduction

**Figure 1:**
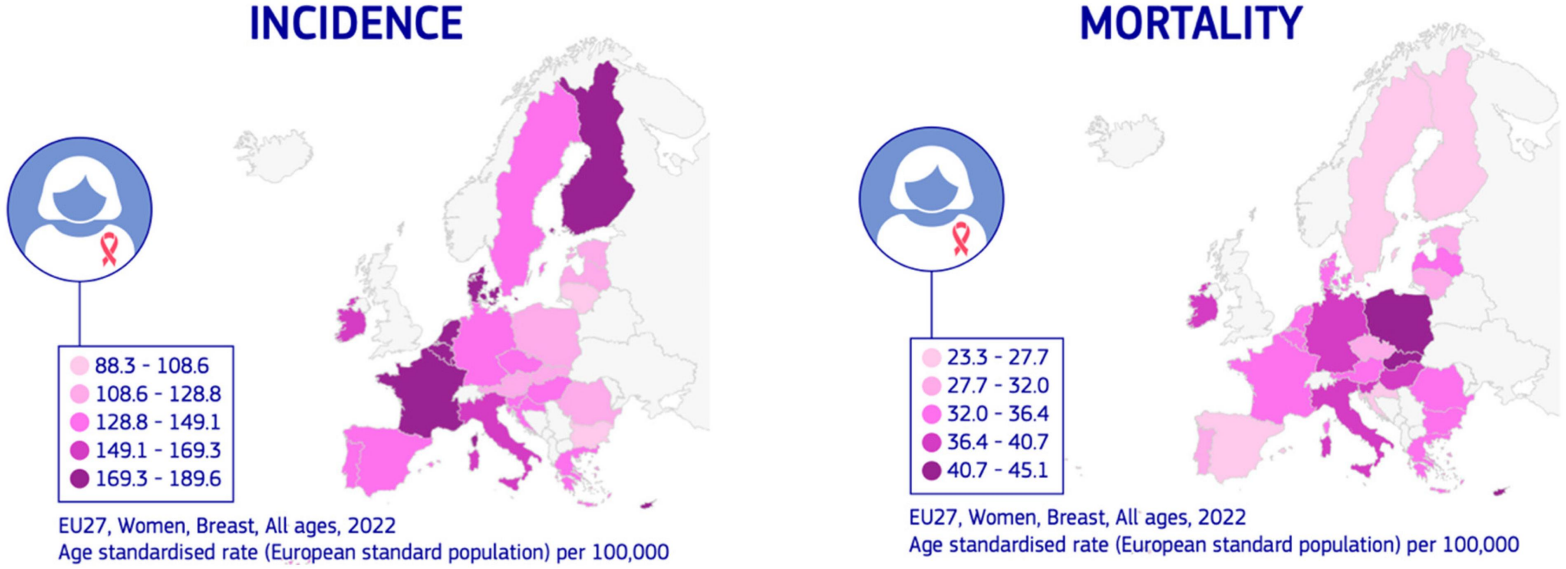
Age-standardised breast cancer incidence and mortality rates among women in the EU-27 in 2022. (https://ecis.jrc.ec.europa.eu/sites/default/files/2024-01/jrc_Breast_cancer_2022_Oct_2023.pdf)

Breast cancer remains one of the most significant public health challenges affecting women worldwide. Globally, it is the most commonly diagnosed cancer in women and accounts for approximately one in four annual cancer cases among women. In 2022, an estimated 2.3 million women were diagnosed with breast cancer worldwide, and approximately 670,000 died from the disease (1). The burden is also substantial in Europe. In the WHO European Region, breast cancer is the most common cancer in women, with an estimated incidence of 604,900 cases in 2022 (1, 2). Within the EU-27, approximately 375,000 new breast cancer cases were estimated in 2022, with a lifetime risk of approximately 1 in 11 women developing the disease before age 74 (2). These global and European figures underscore the continued importance of effective prevention, early detection, and risk-adapted screening strategies. Table 1 and Figure 1 summarize the breast cancer burden across the EU-27, the WHO European Region, and the five focal countries included in this study.

**Table 1:** Estimated breast cancer incidence and mortality in 2022 for the EU-27, the wHO European region, and the five focal countries included in this study.

Country/Region	Incidence		Mortality	
	Numbers	Age-standardised rate (W)	Numbers	Age-standardised rate (W)
EU-27	375,079	83.4	95,881	14.9
WHO Europe region	604,941	70.7	160,043	14.5
Estonia	857	63.1	267	13.2
France	65,659	105.4	14,739	15.8
Portugal	8,954	88.8	2,211	14.5
Spain	34,735	81.5	6,747	10.6
Sweden	7,452	81.4	1,535	11.9

Age-standardised rate (W): A rate is the number of new cases or deaths per 100,000 persons per year. An age-standardised rate is the rate that a population would have if it had a standard age structure. Standardization is necessary when comparing several populations that differ with respect to age because age has a powerful influence on the risk of cancer. WHO Europe region includes 53 countries. Incidence data include all countries except for Andorra, Monaco and San Marino.

Most organized breast cancer screening programs remain primarily age-based, commonly inviting women from around age 50 for routine mammography. Although this approach has contributed to earlier detection at the population level, it does not fully account for differences in inherited, familial, or polygenic risk. Approximately 5%–10% of breast cancers are considered hereditary, meaning they are directly linked to inherited pathogenic variants such as BRCA1, BRCA2, or other breast cancer susceptibility genes (3). However, inherited susceptibility is not limited to rare high-penetrance variants. Some women may have elevated risk due to family history, moderate-risk variants, or the cumulative effect of many common genetic variants (4–7). As a result, younger women who are outside routine screening age but have elevated genetic risk may be overlooked, potentially contributing to delayed diagnosis, later-stage detection, missed opportunities for earlier surveillance, and less individualized prevention counselling (8–11). Identifying elevated inherited risk may also be relevant for family members, who may benefit from genetic counselling, risk assessment, cascade testing where appropriate, and tailored prevention or screening recommendations (10, 11).

Polygenic risk scores (PRS) have emerged as a promising tool for refining breast cancer risk assessment. PRS estimate risk by aggregating the effects of many common genetic variants, thereby complementing rather than replacing existing approaches such as age-based screening, family history assessment, and monogenic testing (4, 5, 12, 13). Compared with age-based screening alone, PRS may improve risk stratification by identifying women whose risk is substantially above or below the population average. Compared with monogenic testing, PRS may capture clinically relevant inherited susceptibility among women who do not carry rare high-penetrance pathogenic variants (4–7). In prevention and early detection, this information may support more personalized screening pathways, including earlier mammography referral, modified screening intervals, additional imaging for selected high-risk groups, or targeted prevention counselling (10, 11, 14–16).

Despite this promise, the implementation of PRS-based breast cancer screening remains complex. Its integration into routine care requires not only scientific validity, but also clinical guidelines, reimbursement mechanisms, professional training, genetic literacy, interoperable digital infrastructure, risk communication tools, and public trust (10–13, 17). These requirements vary substantially across European health systems, where governance structures, screening programs, health technology assessment pathways, and digital health infrastructures differ (18, 19). Understanding these differences is essential for assessing whether and how PRS-based screening can move from pilot projects to routine public health practice.

The BRIGHT project provides an opportunity to examine these implementation dynamics across different European health system contexts. This study investigates how PRS-based breast cancer screening is being piloted, adapted, and resisted across Estonia, Portugal, Spain, Sweden, and France (20–22). By comparing these countries, the study aims to identify common barriers, facilitators, and strategic pathways for integrating PRS into breast cancer prevention and screening.

### 1.1 Conceptual background and analytical frameworks

This study draws on complementary conceptual frameworks to examine PRS implementation as both a clinical innovation and a health-system transformation process. First, the Value–Information–Process (VIP) framework is used to examine how stakeholders define the value of PRS-based screening, how genetic and clinical information is generated and exchanged, and how implementation processes are organized across institutions (23). Second, selected constructs from the Consolidated Framework for Implementation Research (CFIR) are used to situate these dynamics within broader outer and inner settings, including policy environments, reimbursement structures, organizational capacity, professional norms, and implementation processes (24, 25). Finally, business model thinking is used to consider how PRS-based screening can be aligned with sustainable service delivery, funding, partnerships, and workflow integration (23, 26). Presenting these frameworks together allows the study to analyze PRS implementation not only as a question of clinical utility, but also as a matter of institutional readiness, information infrastructure, and governance alignment.

## 2 Materials and methods

### 2.1 Study design and case selection

This study used a qualitative, multiple-case study design to examine the implementation of polygenic risk score (PRS)-based breast cancer screening across different European healthcare systems. The analysis focused on five country cases: Estonia, Portugal, Spain, Sweden, and France. These countries were selected because they offered contrasting implementation contexts in relation to health-system governance, digital infrastructure, genomic medicine readiness, reimbursement pathways, and PRS-related policy or pilot activity (20–22, 27).

The inclusion criteria for focal country cases were: (1) relevance to PRS-based breast cancer screening implementation or policy development; (2) evidence of pilot activity, clinical interest, or institutional readiness for PRS-based screening; (3) variation in health-system governance and implementation pathways; and (4) availability of sufficient empirical or documentary material to support case development. Countries were excluded from focal case analysis if the available data were too limited to support a full country case, if PRS-based breast cancer screening was not sufficiently relevant to the national context, or if interviews served mainly to provide contextual rather than case-specific information.

Primary empirical data were collected through interviews in Estonia, Sweden, Portugal, and Spain. France was included as an analytically derived comparator case because of its relevance to PRS implementation, particularly its national genomics infrastructure, formalized health technology assessment system, and the MyPeBS trial (28–30). However, primary interviews could not be completed in France during the study period because access to appropriate stakeholders was limited and recruitment did not yield sufficient participation before data collection closed. The French case was therefore developed using policy documents, published literature, trial materials, project outputs, and comparative interpretation.

Although interviews were conducted with stakeholders in additional countries, these interviews were not treated as primary case studies. Instead, they were used to contextualize broader European and international implementation dynamics. These countries were not developed as full cases because the data did not provide sufficient country-level depth, stakeholder diversity, or direct relevance to the BRIGHT implementation settings.

### 2.2 Stakeholder identification and sampling

Participants were identified through purposive and snowball sampling. Initial stakeholder mapping was conducted in each focal country to identify individuals and organizations involved in breast cancer screening, genomic medicine, health technology assessment, reimbursement, regulation, patient advocacy, diagnostics, and PRS-related implementation. This mapping drew on BRIGHT consortium knowledge, national policy documents, institutional websites, scientific publications, professional networks, and recommendations from early informants (20–22, 27, 31).

Stakeholders were categorized according to their primary function in the PRS implementation pathway rather than by job title alone. The main stakeholder categories were: (1) technical and economic decision-makers, including representatives of health ministries, HTA bodies, reimbursement agencies, and public payers; (2) medical professionals and key opinion leaders, including oncologists, radiologists, gynecologists, primary care physicians, hospital managers, screening experts, and researchers involved in cancer prevention or genomic medicine; (3) patient representatives and advocacy organizations; (4) regulatory authorities, notified bodies, or actors involved in diagnostic and *in vitro* diagnostic oversight; and (5) healthy women aged 35–49, corresponding to the target population for PRS-based screening approaches.

Eligible participants were adults with professional, policy, advocacy, regulatory, industry, research, or target-user relevance to PRS-based breast cancer screening, breast cancer prevention, genomic medicine, or screening governance. Individuals were excluded if they had no relevant connection to breast cancer screening, PRS, genomic medicine, health technology assessment, prevention policy, or the target population. Potential participants were contacted by email, provided with information about the study, and invited to participate in a semi-structured interview. Additional participants were identified through snowball referrals from interviewees and project partners.

### 2.3 Data collection

Data collection included semi-structured interviews and document analysis. Interviews were conducted between October 2022 and March 2024. Interviews lasted approximately 45–90 min and were conducted either in person or through secure video-conferencing platforms. With participant consent, interviews were audio-recorded, transcribed verbatim, translated into English where necessary, and anonymized before analysis.

The interview guide was informed by the study's analytical frameworks and focused on the perceived value, information requirements, and implementation processes associated with PRS-based breast cancer screening (23, 24). All participants were asked a common set of core questions covering: current breast cancer screening pathways; perceived value and limitations of PRS; expected benefits and risks; implementation barriers and facilitators; data flows and digital infrastructure; reimbursement and HTA requirements; professional roles and training needs; genetic literacy; risk communication; stakeholder responsibilities; and conditions for scale-up. Stakeholder-specific probes were used depending on participants' expertise. For example, policymakers and payers were asked more detailed questions about reimbursement, HTA, and governance; clinicians were asked about workflow integration, counselling, and clinical decision-making; patient representatives were asked about acceptability, trust, and communication; and healthy women were asked about perceived usefulness, concerns, and willingness to participate in risk-based screening.

Document analysis was used to contextualize and triangulate interview findings. Documents included national genomics strategies, cancer screening policies, HTA and reimbursement materials, clinical and policy reports, pilot-study documentation, published trial protocols, and publicly available materials on PRS-related initiatives (11, 20–22, 27–31). Documents were identified through targeted searches, consortium expertise, references provided by interviewees, and review of national institutional sources. Relevant information was extracted on governance arrangements, reimbursement pathways, screening infrastructure, digital health capacity, pilot initiatives, and implementation barriers. These data were compared with interview findings during case development and cross-case synthesis. For France, document analysis and published evidence formed the primary basis of the analytically derived case (28–30).

### 2.4 Data analysis

Interview transcripts and documentary materials were analyzed using a combination of inductive thematic analysis and deductive framework mapping. NVivo 14 was used to support data organization and coding. Initial coding identified recurring themes related to PRS value, implementation barriers, stakeholder roles, information flows, governance structures, reimbursement, professional readiness, and risk communication. These themes were then mapped to the Value–Information–Process framework and selected constructs from the Consolidated Framework for Implementation Research, as described in the conceptual framework section (23, 24).

Each country case was first analyzed individually to identify country-specific implementation conditions, barriers, facilitators, and strategic entry points. Cross-case analysis was then conducted to compare implementation patterns across countries and to identify common and divergent pathways. Data triangulation was achieved by comparing interview findings with policy documents, pilot evidence, and stakeholder presentations (11, 17, 20–22, 27–31). Preliminary interpretations were shared with 14 key interviewees across three countries to validate findings and refine country-level interpretations.

### 2.5 Researcher reflexivity and trustworthiness

The research team included investigators with backgrounds in health policy, implementation science, oncology, genomics, health economics, and healthcare management. Several authors were involved in the BRIGHT project, which provided access to relevant stakeholders and detailed contextual knowledge, but also created the possibility of assumptions favoring PRS implementation. To address this, the analysis combined inductive coding with deductive framework mapping, triangulated interviews with documentary evidence, and incorporated regular research team discussions to challenge emerging interpretations.

Trustworthiness was strengthened through multiple strategies. First, data were collected from diverse stakeholder groups across different health-system contexts. Second, interview findings were triangulated with national policy documents, pilot materials, and published evidence. Third, preliminary interpretations were shared with selected key interviewees for member-checking. Full transcripts were not routinely returned to participants for correction; instead, validation focused on country-level interpretations and emerging findings. Fourth, anonymization and team-based discussion were used to reduce the influence of individual researcher assumptions on interpretation.

### 2.6 Ethical considerations

Ethical approval was obtained from the coordinating academic institution, IESE Business School, Spain, in accordance with the principles of the Declaration of Helsinki, and confirmed with institutional partners where required. All participants received information about the study before participation and provided informed consent. Participants were informed of their right to withdraw at any time and were assured that data would be anonymized and reported confidentially.

## 3 Results

This section presents five country cases: Estonia, Portugal, Spain, Sweden, and France, describing the institutional, regulatory, and organizational contexts in which PRS-based breast cancer screening is being explored or considered. The results draw on interview data, policy documents, and evidence from relevant PRS-related pilot initiatives. Interview findings were used as the primary empirical source for Estonia, Portugal, Spain, and Sweden, while documentary and published evidence were used to contextualize all cases and to develop the analytically derived French case. The interview sample included stakeholders from several broad categories relevant to PRS-based breast cancer screening, including technical and economic decision-makers, medical professionals and key opinion leaders, patient representatives, regulatory or diagnostic actors, and healthy women aged 35–49. Table 2 summarizes the distribution of participants by country and stakeholder category. Because professional subgroups were not recorded in a fully standardized way across all countries, the analysis reports stakeholder composition at the broader category level rather than by specific professional title. This approach avoids overinterpretation while still indicating the range of perspectives represented in the five focal cases. Cross-case comparisons and interpretive synthesis are presented after the country cases.

**Table 2:** Interview participants by country and stakeholder category.

Country	Technical & Economic Buyers	Medical Professionals/KOLs	Healthy Women/Patients	Patient Organizations	Regulatory/Notified Bodies	Total Interviews
Estonia	6	18	19	0	0	43
Sweden	5	11	2	3	4	25
Portugal	2	1	1	2	0	6
Spain	5	3	0	0	1	9
UK	0	3	0	0	0	3
Switzerland	0	1	0	0	0	1
Brazil	0	1	0	0	0	1
US	0	2	0	0	0	2
Netherlands	0	1	0	1	0	2
Germany	0	1	0	0	0	1
Belgium	0	1	0	0	0	1
Total	18	43	22	6	5	94

### 3.1 Spain

Spain presents a complex implementation landscape for PRS-based breast cancer screening, shaped by a highly decentralized healthcare system, where the 17 autonomous communities (CC.AA) maintain authority over health service delivery, funding, and technological adoption. While the national Ministry of Health establishes baseline standards through the Common Portfolio of Services, regional variation remains significant, which stakeholders described as creating both opportunities and barriers for health innovation. The 2024 rollout of the *Common Catalog of Genetic and Genomic Tests*, under the Recovery, Transformation, and Resilience Plan (Component 18), marked a major policy milestone, supported by €50 million in public investment. Although PRS is not yet included in the catalog, interviewed stakeholders anticipated that PRS may gain future relevance, particularly in oncology. The law requires HTA to consider the efficacy, cost, efficiency, effectiveness, safety, and therapeutic utility of the different alternatives. However, there are no national or regional guidelines related to data requirements for HTA. The data used in each HTA evaluation is decided case by case by the HTA agencies.

**Figure 2:**
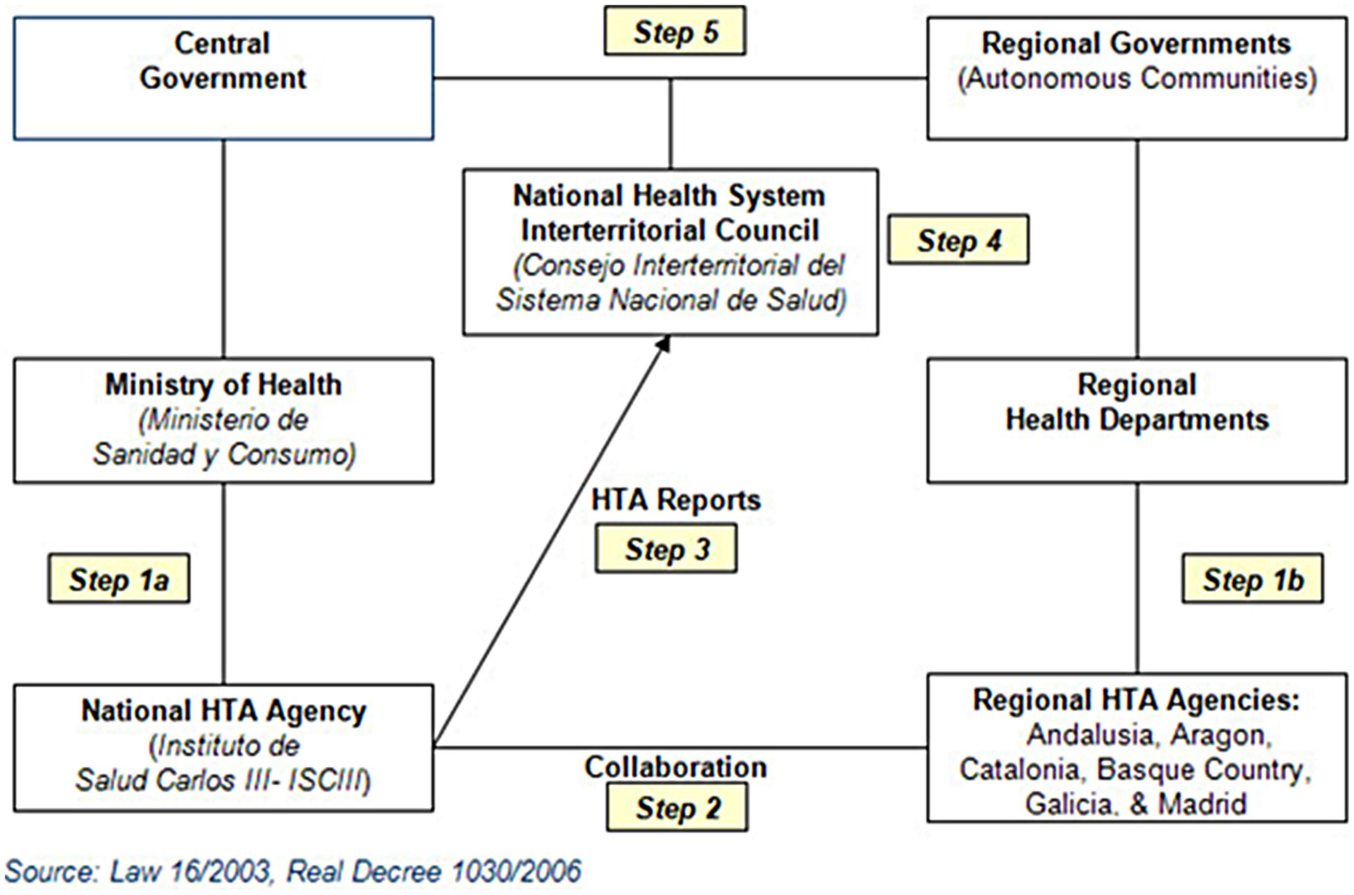
HTA process for decision-making related to the national catalogue of services in Spain. The central and regional governments request HTA evaluations from the national and regional agencies (Steps 1a and 1b). The ISCIII and regional agencies collaborate to produce the required HTA information (Step 2). The ISCIII produces the HTA report and submits it to the Interterritorial Council (Step 3). The Interterritorial council decides about the inclusion or exclusion of technologies in the national catalogue (Step 4). The decision of the Interterritorial council is implemented by the central and regional governments (Step 5).

Pathways for implementation are currently threefold: (1) national adoption via the Interterritorial Council (Figure 2); (2) regional-level inclusion into local benefit packages, as seen in proactive CC.AA like Catalonia, Madrid, and the Basque Country; and (3) hospital-level procurement led by clinicians and departments using institutional budgets.

Catalonia has emerged as the frontrunner in PRS adoption, leveraging its Oncology Master Plan 2020–2025, which prioritizes precision medicine and the integration of real-world data. The *Comissió Assessora de Cribratge de Càncer* has experience incorporating risk-stratified approaches, having already adapted cervical cancer screening protocols. The DECIDO study (27), a proof-of-concept trial conducted in Catalonia, provided early evidence on the feasibility, acceptability, and logistical requirements for personalized breast cancer screening. It revealed favorable attitudes toward more intensive screening for high-risk women, while reduced screening frequency for low-risk groups was met with skepticism, especially among less-educated populations. Health professionals highlighted systemic challenges, including the lack of a formal genetics specialty (Spain remains the only EU country without one), low genetic literacy among primary care professionals, and IT limitations in population risk communication. Nevertheless, facilitators such as women's proactive engagement, trust in primary care, and civil society support (e.g., La Liga Contra el Cáncer) offer fertile ground for localized pilots.

Despite regional advancements, national integration of PRS within Spain remains uncertain. Inclusion in the Common Catalog requires evidence of clinical utility and cost-effectiveness, typically assessed by the Instituto de Salud Carlos III in collaboration with regional HTA bodies. The absence of a centralized mechanism for modifying screening programs contributes to slow national-level integration. In parallel, private sector actors are currently offering PRS tests in selected clinical settings. For BRIGHT, Spain's landscape underscores the importance of multi-level engagement, simultaneously building evidence for national reimbursement while leveraging regional innovation ecosystems and hospital-driven pilots as immediate entry points.

### 3.2 Portugal

**Figure 3:**
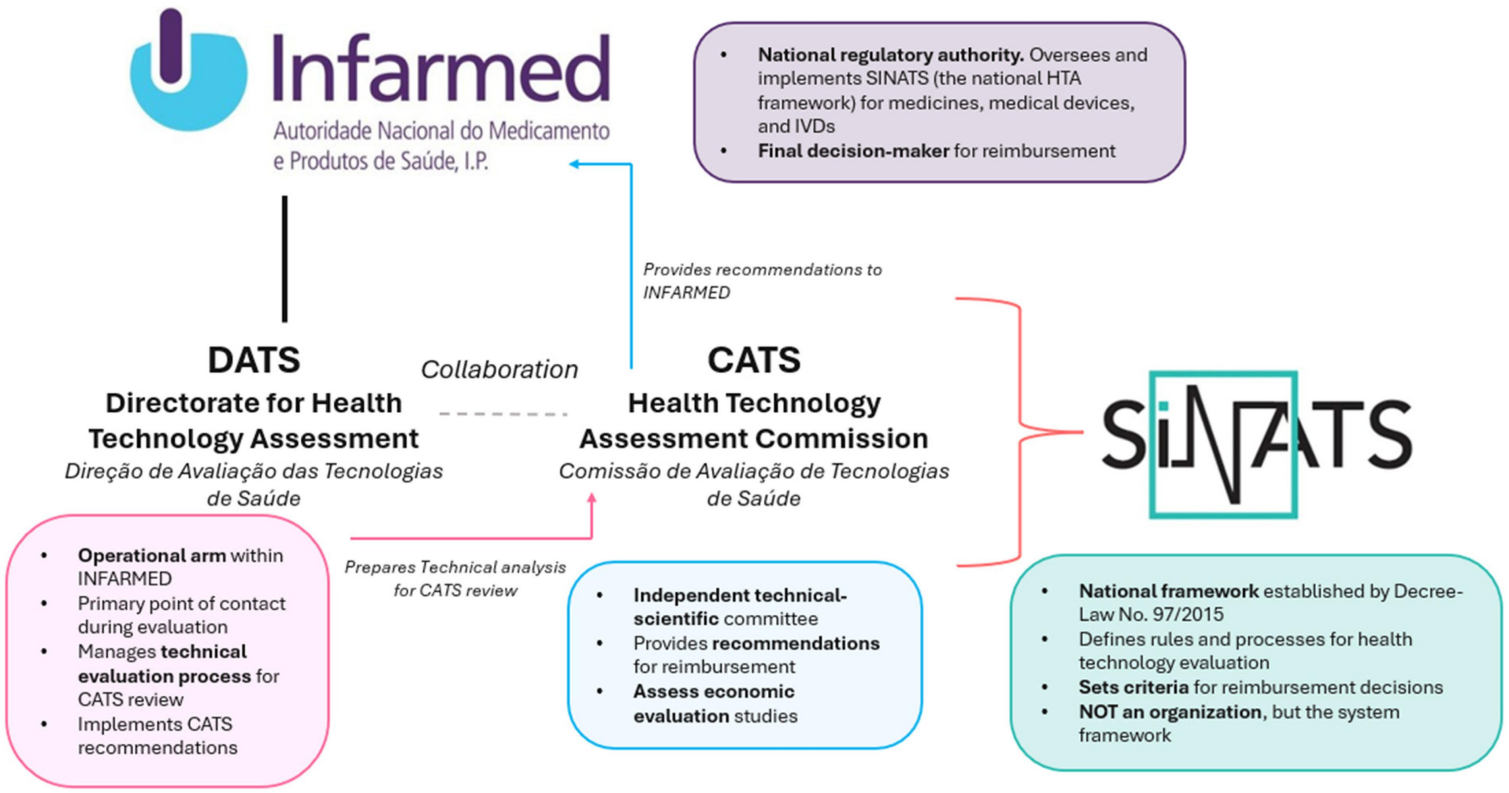
Outlines the institutional landscape for health technology assessment and reimbursement in Portugal, detailing the roles of INFARMED, DATS, and CATS within the SINATS framework.

Portugal presents a centralized but risk-averse environment for the implementation of PRS-based breast cancer screening. Oversight of health services lies with the Ministry of Health, while INFARMED, the National Authority of Medicines and Health Products, regulates the approval and reimbursement of new technologies through a rigorous multi-step process. This process is structured around two core institutions: the Directorate for Health Technology Assessment (DATS), which serves as the operational and technical arm, and the Health Technology Assessment Commission (CATS), an independent committee responsible for issuing scientific recommendations on reimbursement. Together, they operate under the SINATS framework (Sistema Nacional de Avaliação de Tecnologias de Saúde), established by Decree-Law No. 97/2015, which defines evaluation criteria such as clinical benefit, economic value, and system-level impact. INFARMED is the final decision-making authority on whether technology is publicly reimbursed (32) (Figure 3).

**Figure 4:**
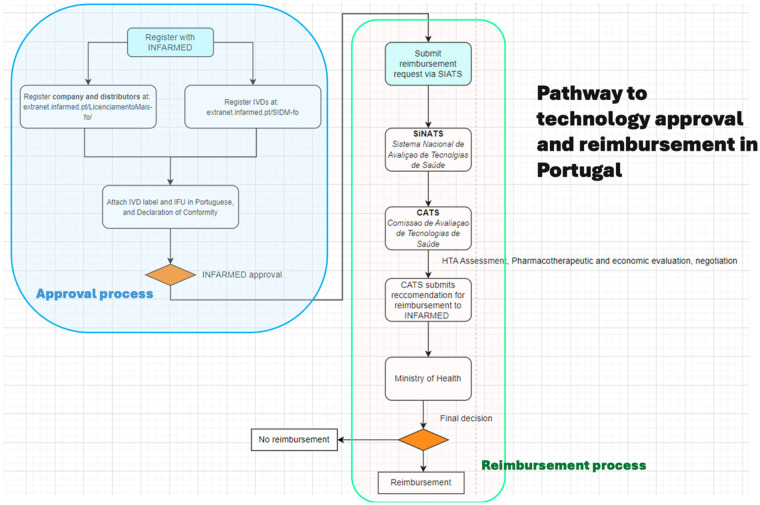
Illustrates the step-by-step approval and reimbursement pathway for *in vitro* diagnostic technologies, like PRS, within the Portuguese healthcare system.

For *in vitro* diagnostics (IVDs) like PRS tests, the process begins with registration through INFARMED's extranet portals, followed by submission of a reimbursement dossier to SINATS. DATS conducts the technical and economic evaluation, CATS reviews the evidence and submits its recommendation, and INFARMED renders the final decision. This linear but multi-actor workflow introduces long lead times and significant uncertainty for innovative tools like PRS. The full approval-to-reimbursement sequence is summarized in Figure 4.

Due to these public sector hurdles, early experimentation with PRS-based services in Portugal is occurring primarily within the private healthcare ecosystem. Leading hospital groups (e.g., CUF, Hospital da Luz, Lusíadas) and diagnostics providers (e.g., SYNLAB, Germano de Sousa, and PALEX) have explored or are in the process of incorporating PRS-based services into executive wellness or preventive care portfolios. These initiatives remain heterogeneous in scope and maturity. They leverage private-sector autonomy and existing laboratory infrastructures to navigate regulatory timelines more flexibly than the public reimbursement pathway.

At the societal level, civil society's engagement is strong. The EVITA patient advocacy group, active at both national and European levels, supports PRS as a lever for modernizing cancer prevention. While primary care physicians remain the first contact point in the public system, stakeholders suggest that specialized oncology and gynecology clinics, or even direct-to-consumer channels with structured referral pathways, may serve as more practical launchpads for PRS deployment. Barriers include limited genetic literacy among general practitioners, slow HTA cycles, and the absence of a recognized clinical genetics specialty. Yet, momentum is building: key opinion leaders such as Prof. Luis Costa have voiced support for pilot studies, and partnerships with the Liga Portuguesa Contra o Cancro are under discussion as part of BRIGHT's next-phase strategy to test hybrid implementation models that bridge clinical innovation and policy uptake.

### 3.3 Sweden

**Figure 5:**
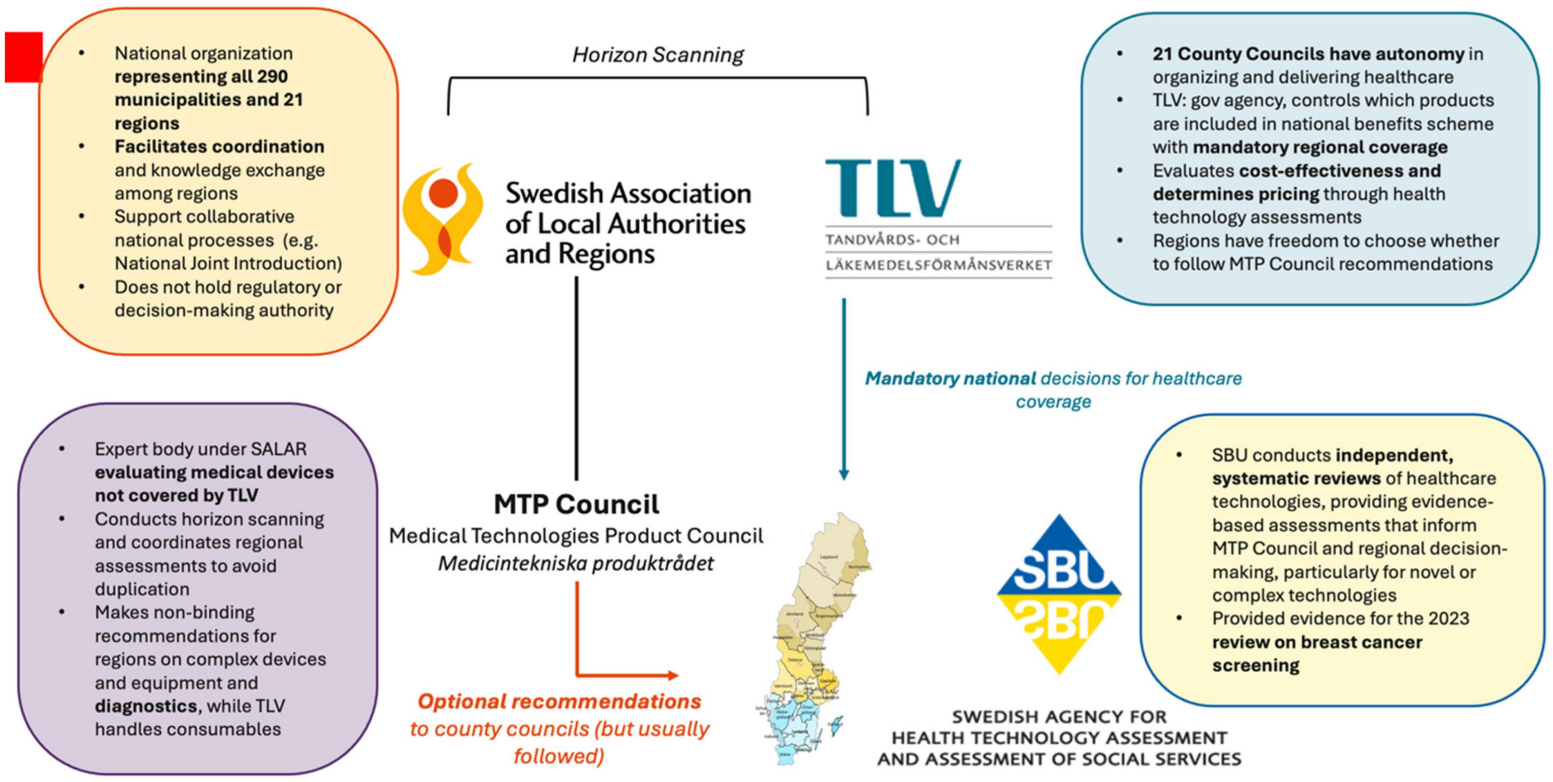
Maps the key national and regional institutions involved in health technology assessment and screening governance in Sweden.

Sweden presents an institutionally complex governance landscape for PRS-based breast cancer screening, shaped by a publicly funded but highly decentralized healthcare system in which 21 autonomous regions are responsible for organizing and delivering health services, including population screening programs. At the national level, strategic coordination occurs through the National Joint Introduction for medical technologies, a collaborative process among regions. Within this framework, the Medical Technologies Product Council (MTP Council) supports regional decision-making through horizon scanning, procurement coordination, and guidance on emerging medical technologies. National reimbursement decisions for pharmaceuticals and selected consumables fall under the Dental and Pharmaceutical Benefits Agency (TLV), while the Swedish Agency for Health Technology Assessment and Assessment of Social Services (SBU) provides independent evidence reviews to inform policy and regional decisions (Figure 5).

**Figure 6:**
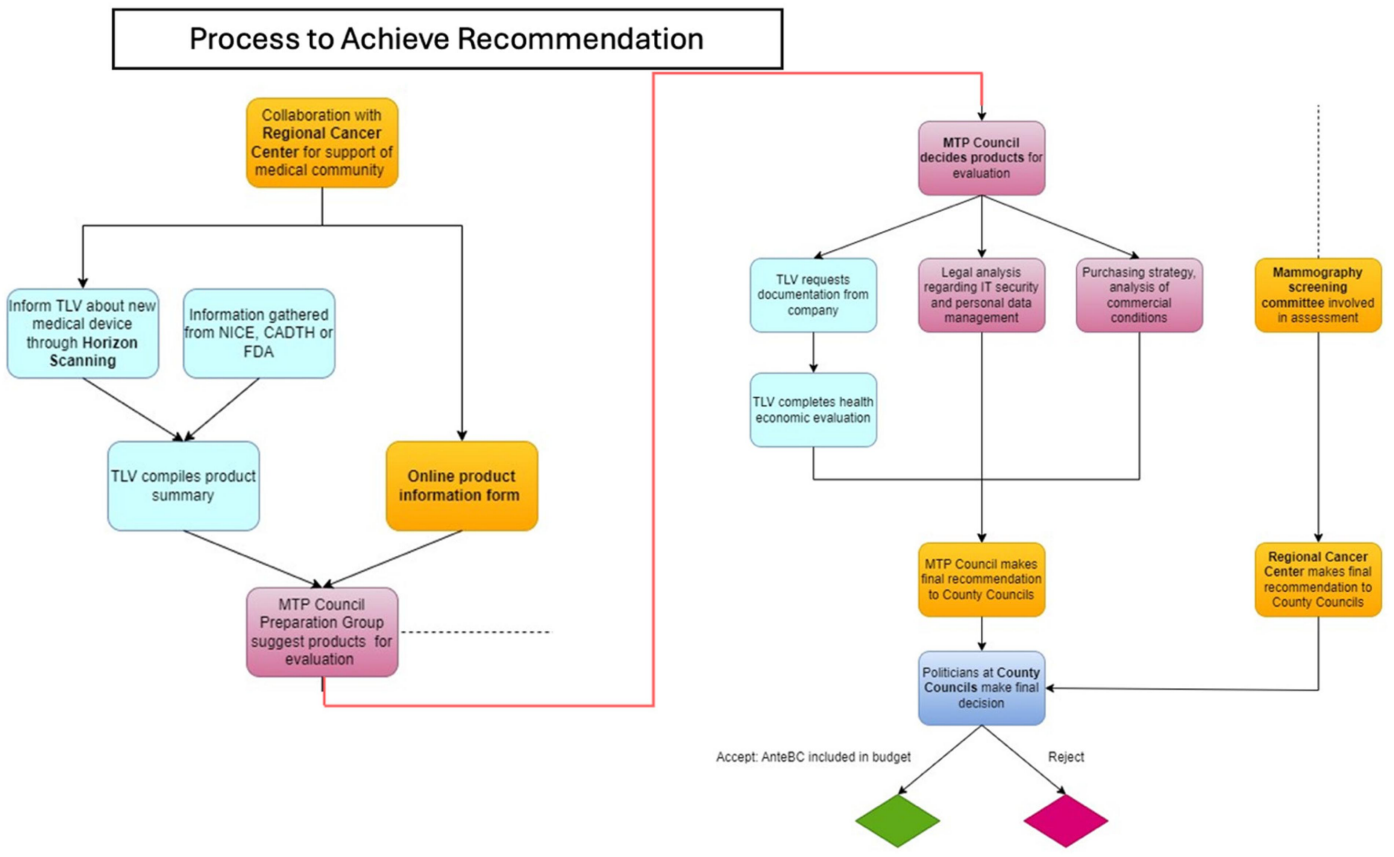
Illustrates the evaluation and decision-making pathway for integrating new diagnostic technologies, such as PRS, within Sweden's decentralized healthcare system.

PRS implementation is likely to follow a regional pilot logic, where individual regions initiate trials or adopt innovations based on local readiness and partnerships. Notably, Region Uppsala, with support from U-CAN (Uppsala/Umeå Comprehensive Cancer Consortium) and academic cancer centers, has positioned itself as a potential first mover in PRS-enabled screening. Decision-making for such initiatives typically follows a coordinated process involving MTP Council input, health economic assessment from TLV, legal review (e.g., data privacy, IT infrastructure), and final approvals by county politicians. The decision-making flow, from horizon scanning to county-level budget allocation, is captured in Figure 6.

Stakeholder interviews across regions confirmed widespread interest in PRS as a modernization tool for breast cancer prevention, particularly due to Sweden's robust biobank infrastructure, digital health ecosystem, and high screening participation rates. However, implementation faces several barriers: (1) lack of a formal reimbursement category for polygenic tests; (2) variability in digital integration capacity across regions; (3) conservative approach to altering mammography workflows; and (4) professional hesitancy stemming from limited training in genetic risk communication. Interviewees emphasized the importance of structured clinician education, automated clinical decision support, and tailored public messaging to ensure equity and mitigate confusion, especially if low-risk women receive recommendations for less intensive screening. Additionally, including PRS in clinical guidelines is very important.

Despite these challenges, Sweden is considered a technically well-prepared environment for PRS piloting in Europe, with strong biobank infrastructures and digital health capabilities, although implementation readiness varies across regions. The BRIGHT consortium is engaging with regional stakeholders to co-design a demonstration project that leverages existing screening infrastructure and positions PRS as a low-friction enhancement to existing workflows rather than a disruptive overhaul. This approach aligns with Sweden's strategic preference for evidence-driven, regionally tested, and nationally guided innovation in healthcare.

### 3.4 Estonia

**Figure 7:**
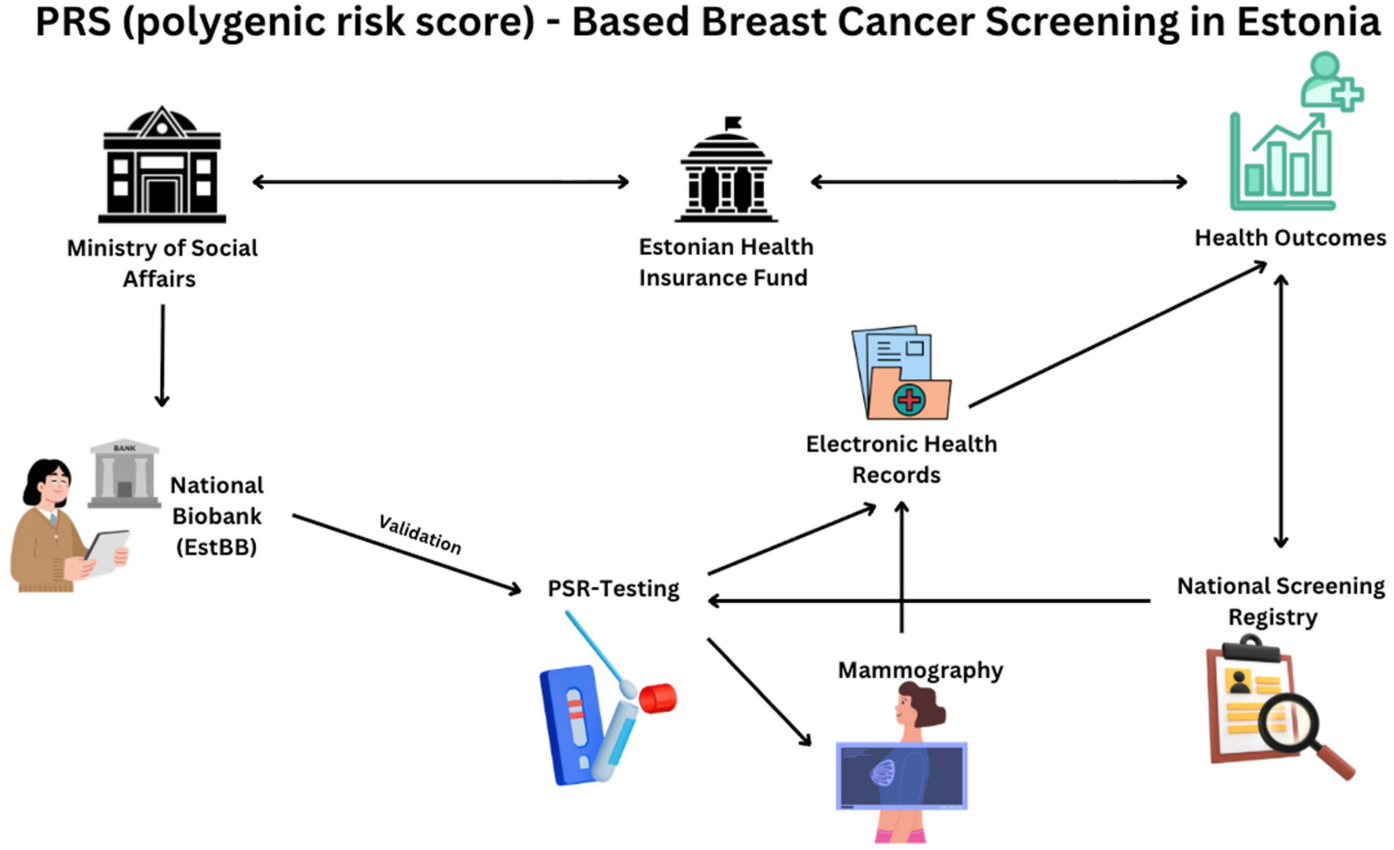
Illustrates how Estonia's digitally integrated health system connects the Estonian biobank, electronic health records, and national institutions to enable the implementation of PRS-based breast cancer screening. The Ministry of Social Affairs establishes the legal and policy framework. The Estonian Health Insurance Fund (EHIF), an independent public legal entity operating under the Ministry of Social Affairs, conducts HTA, approves services and reimbursement, and finances healthcare services while considering health outcomes. The National Screening Registry defines target groups, sends invitations, and analyzes screening outcomes. The Estonian Biobank (EstBB) is used for PRS test validation and provides genotyping data for its participants (gene donors). PRS and mammography data are entered into the electronic health records (EHR). Mammography recommendations are provided by healthcare providers through the Central Health Information System Health Portal.

Estonia provides one of the most conducive environments for implementing PRS-based breast cancer screening in Europe, due to its highly digitalized and centrally coordinated healthcare infrastructure. Strategic governance is led by the Ministry of Social Affairs, while service development, financing and service procurement fall under the Estonian Health Insurance Fund (EHIF). This streamlined structure enables rapid national implementation when innovation aligns with policy priorities and demonstrates cost-effectiveness (Figure 7).

Personalized breast cancer prevention services based on the clinical grade level PRS breast cancer test (AnteBC™ by OÜ Antegenes) and family cancer history have already been introduced in Estonian healthcare as private medical services, but are also followed with mammography screening services for women under the age of 50 (before the start of routine screening), which are funded by the public Estonian Health Insurance Fund based on PRS recommendations (11).

The BRIGHT project launched its most comprehensive pilot in Estonia, focusing on a more complex personalized breast cancer screening service model that includes the PRS test (AnteBC™) (17).The project tested different channels for women's engagement in the service (digital telemedicine, breast clinics, family physician practices, pharmacies) and replacing verbal genetic test pre- and post-test counseling with digital decision support for both patients and healthcare professionals, demonstrating the feasibility of a new standard for personalized screening and prevention based on genetic risks (10).

The pilot benefited from the digital telemedicine platform for personalised breast cancer screening service and from the digital decision support for patients and medical professionals, connected with Estonia's fully integrated Electronic Health Record (EHR) system, which allows seamless interoperability between primary care, specialty care, and biobank-based research platforms (17).

Several enablers facilitated early adoption: (1) centralized procurement and reimbursement capacity; (2) integrated clinical and research data infrastructures via the Estonian Biobank; and (3) a nimble policy process that allows for rapid translation of research into public health practice; (4) already available PRS-based personalised breast cancer prevention services in Estonian private healthcare; (5) developed clinical recommendations for breast cancer PRS clinical use, supported by digital decision support (10, 17). Indeed, by 2022, the EHIF had already begun accepting PRS reports of women younger than screening age as valid indications for mammography referrals within the national screening system, reflecting early real-world integration of genetic risk-based screening approaches. Interviews with officials from the Ministry of Social Affairs, the Estonian Health Insurance Fund (EHIF), and clinical leaders in Tartu and Tallinn indicated broad institutional support for integrating PRS into breast cancer prevention pathways. Stakeholders described PRS as a cost-neutral enhancement to existing screening, particularly for women aged 35–49 who are not covered by routine mammography programs. They also emphasized its alignment with Estonia's national genomics strategy and broader efforts to modernize population health prevention. Building on earlier pilots, Estonia has already initiated nationwide testing of PRS-based breast cancer screening through a population-based implementation study embedded within the public health system, supporting the transition from pilot initiatives toward potential routine integration in the national screening program (22).

Recent developments indicate substantial progress toward the national implementation of PRS-based breast cancer screening in Estonia. Genotyping and PRS-based services have been formally included in the national Health Services List, enabling standardized coding and reimbursement through the Estonian Health Insurance Fund (EHIF) and allowing structured contracting with healthcare providers. In parallel, interoperability between the National Screening Registry and the Electronic Health Record (EHR) platform has been operationalized, enabling secure data exchange for identifying screening target groups and automatically distributing invitations within the organized screening program. The service model planned for implementation in 2026 designates midwives in breast clinics as primary risk-counselling providers. Rather than relying on specialized genetic counsellors, the model emphasizes the use of digital decision-support tools for primary healthcare professionals and patients, providing standardized clinical recommendations for managing different genetic risk levels. Training on polygenic risk communication has previously been delivered to general practitioners through the EstPerMed project; however, periodic updates and refresher training may be required to maintain clinical readiness. Findings from the ongoing national implementation study will further assess provider preparedness and identify potential training needs. Implementation governance has also advanced. The EHIF has convened a clinical advisory working group and commissioned the development of national clinical guidelines for PRS-based breast cancer screening from the University of Tartu. Prior to the program launch, a screening code of conduct will be developed and approved by the Screening Steering Group established at the Ministry of Social Affairs, ensuring alignment between clinical practice, digital infrastructure, and national screening governance.

Estonia represents a leading example for how a digital-first, policy-aligned health system can serve as a launchpad for genomic innovation. The BRIGHT pilot has already demonstrated both technical feasibility and institutional interest, positioning Estonia as a European reference site for scaling risk-based cancer screening (17). Estonia is also currently preparing to adopt PRS testing in its breast cancer public screening program in 2026.

### 3.5 France

France combines one of Europe's most advanced genomics infrastructures with a highly formalized and risk-regulated healthcare system, shaping a complex environment for the implementation of PRS-based breast cancer screening. Anchored by the Genomic Medicine 2025 Plan, a €670 million state-led strategy, France has established cutting-edge sequencing hubs, SeqOIA in Paris and AURAGEN in Lyon, and centralized data integration through the Centre d'Analyse de Données (CAD). These institutions aim to transition next-generation sequencing from rare disease and oncology into broader clinical use, although preventive applications, like PRS, remain on the margins.

National governance for cancer prevention is shared among the Ministry of Health, the National Cancer Institute (INCa), the High Authority for Health (HAS), and Assurance Maladie, France's public insurance fund. While this provides clear accountability, it also imposes strict conditions on innovation uptake, including high evidentiary thresholds and approval via HTA pathways governed by CNEDiMTS (for clinical relevance) and CEESP (for economic evaluation). Inclusion in the national benefits package (LAP – Liste des Actes et Prestations) remains a key bottleneck, as PRS is not yet formally reimbursed. Direct-to-consumer testing is prohibited under French bioethics law, which restricts all genetic risk communication to clinical contexts delivered by certified geneticists or authorized oncology centers.

Despite this, France is home to one of Europe's most influential PRS pilot projects, MyPeBS (My Personal Breast Screening), coordinated by Unicancer and funded by the Horizon 2020 program. MyPeBS compares standard mammography to risk-adapted screening based on clinical, familial, and genomic data, using MammoRisk™, a CE-marked PRS platform developed by Predilife (28, 29). The trial has seen strong engagement from mutual insurers (e.g., CNAM, MGEN) and participating clinicians, and is supported by structured physician training and communication protocols. Documentary evidence and BRIGHT project materials suggest that MyPeBS has increased attention to PRS among French clinicians and patients; however, broader integration remains challenged by unclear reimbursement channels, absence of national PRS guidelines, and hesitation from INCa and ARS to deviate from population-level screening models (30).

**Figure 8:**
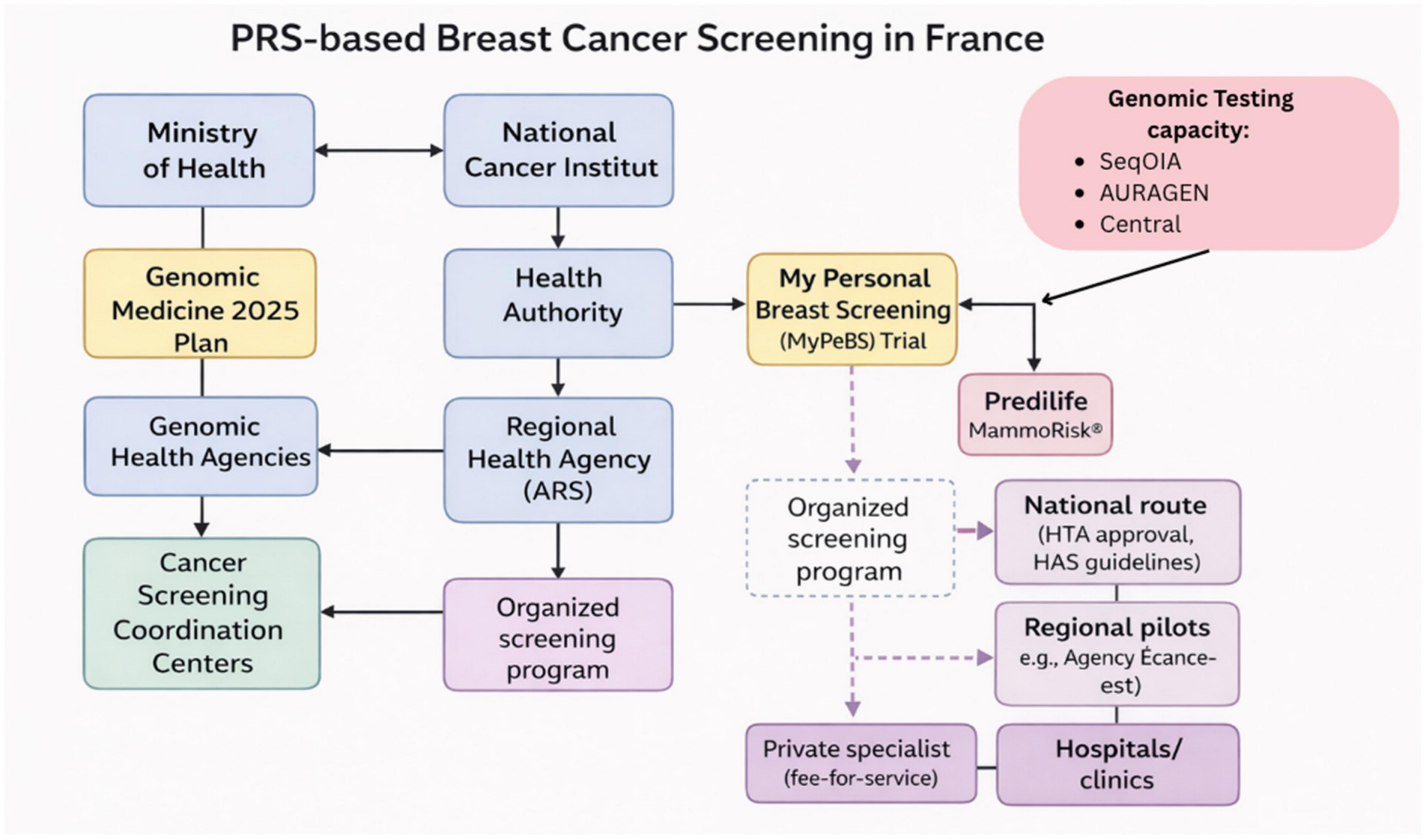
This diagram illustrates the institutional landscape and implementation pathways for PRS-based breast cancer screening in France. It maps key actors, from national genomic initiatives to screening authorities, and highlights both public and private routes for integration.

France's institutional configuration offers several potential implementation pathways (Figure 8). First, a national route through INCa and HAS, contingent upon HTA endorsement and economic modeling, could lead to PRS integration into the organized screening program (Dépistage Organisé du Cancer du Sein). Second, regional pilots under the authority of ARS (Agences Régionales de Santé) provide decentralized opportunities for localized testing, particularly in Paris and Lyon, where infrastructure and political capital already exist. Finally, the private specialist route, where PRS is offered within hospital settings or semi-private centers through fee-for-service models, presents a viable B2C/B2B entry point, albeit with equity concerns.

Applying the VIP framework, PRS in France is seen as offering high value among policy experts, especially when aligned with the country's long-term prevention goals, but remains constrained in terms of process integration (slow HTA cycles, rigid screening protocols) and information dissemination (fragmented EHR systems, low public genetic literacy). Stakeholders agreed that PRS would likely need to “piggyback” on existing trials or disease-area platforms (e.g., hereditary cancer pathways or monogenic screening) to gain traction in the national agenda (23).

In summary, France is emblematic of a “technically ready, institutionally gated” system: sequencing capacity and professional expertise are strong, yet implementation is hampered by legacy regulatory structures, reimbursement inertia, and limited room for preventive genomic innovation. The BRIGHT project positions MyPeBS as a pivotal case, not only in terms of feasibility, but as a trigger for wider system dialogue on integrating PRS into national cancer control policies.

### 3.6 Cross-case analysis using the VIP framework and CFIR

This chapter synthesizes findings across five national cases, Estonia, Portugal, Spain, Sweden, and France, to identify common patterns and divergences in the implementation of PRS-based breast cancer screening. Building on the Value–Information–Process (VIP) framework, we map shared enablers, contextual barriers, and institutional logics that shape readiness for PRS adoption (24, 25). Rather than retelling each case, this analysis draws out comparative insights that inform broader implementation strategy and policy alignment.

We complement the VIP lens with selected dimensions from the Consolidated Framework for Implementation Research (CFIR) (23, 33), particularly outer and inner settings (e.g., national reimbursement pathways, institutional digital maturity) and process mechanisms (e.g., piloting, professional engagement).

Table 3 summarizes the five countries' implementation profiles, clustering them by governance type, dominant PRS value discourse, and observed strategic entry points.

**Table 3:** Countries' implementation profiles.

Country	System Type	PRS Readiness Level	Dominant Value Logic	Strategic Entry Point
Estonia	Centralized, public-private mix, digital-first	High	Efficiency & digital health leadership	National purchase by EHIF, EHR integration. Biobank as supporting genetic data source.
Portugal	Centralized, public-private mix	Medium	Innovation prestige (private sector-led)	Private hospitals and insurance-backed models
Spain	Decentralized (17 CC.AA)	Medium	Cost-effectiveness & regional autonomy	Regional pilots (e.g., Catalonia's DECIDO)
Sweden	Regionalized (21 regions)	Medium	Patient equity & institutional legitimacy	Regional Cancer Centers (RCC)-driven pilots and national coordination
France	Centralized, risk-regulated	Medium	Clinical conservatism & HTA rigor	National trials (MyPeBS) + future LAP inclusion

#### 3.6.1 Value

Stakeholders across all five countries recognized the potential value of PRS-based breast cancer screening, but the framing of that value, what it is, who it benefits, and how it is justified, varied significantly. These differences shaped strategic support, policy traction, and resource mobilization. In Estonia, value was framed in terms of system efficiency and digital modernization. Policymakers and biobank leaders saw PRS as a low-cost enhancement that could better target screening and reduce unnecessary interventions, aligning with the country's long-standing genomic and digital health priorities. In Sweden, value centered on equity and legitimacy: PRS was seen as a way to offer more personalized and justifiable screening for younger women, but only if it could be equitably scaled and governed through public institutions. In contrast, Portugal exhibited a more innovation-driven discourse, particularly among private sector actors who emphasized market differentiation and reputational value, while public stakeholders remained more risk-averse. In Spain, the value narrative was cost-effectiveness-focused, especially in regions like Catalonia, where economic modeling from DECIDO and stakeholder buy-in positioned PRS as a rational extension to stratified public screening. France combined high-level biomedical ambition (Genomic Medicine 2025) with clinical conservatism, framing value as contingent on evidence, institutional consensus, and alignment with established cancer plans. Across all cases, stakeholders emphasized that demonstrating both clinical utility and system-level return, especially through pilot results, was critical to sustaining the perception of PRS as a “value-adding” rather than disruptive technology.

#### 3.6.2 Information

Information systems, including both technological infrastructure and human capacity to interpret risk, were foundational to PRS implementation across settings. Estonia was a clear outlier in terms of digital readiness: the implementation and development of PRS-based services within private healthcare, nationwide EHR, linked biobank infrastructure, and government-supported genomics policy enabled streamlined data collection, storage, and actionable reporting. Stakeholders reported minimal friction in PRS data interpretation, aided by standardized referral logic embedded in clinical software. Sweden followed closely, with advanced biobank integration and regionally harmonized IT platforms managed through RCCs. However, fragmented access to genetic counseling and varying IT literacy among primary care professionals created uneven data use. Spain and Portugal faced more structural constraints: while regions like Catalonia invested in interoperable health platforms, most screening programs operated through non-integrated registries, requiring manual uploads and limited cross-institutional visibility. In Portugal, PRS information largely circulated in the private sector, often outside national registries or EHRs, raising concerns about data fragmentation. France had world-class sequencing capacity (via SeqOIA and AURAGEN), but faced structural siloes: PRS was excluded from national data systems, and stakeholders described limited information flow between trial-based pilots (e.g., MyPeBS) and public health decision-makers. Across all five countries, genetic literacy among frontline providers, especially GPs, was a widely cited bottleneck, reinforcing the need for interpretation tools, decision-support software, and patient education materials to make PRS data usable, not just accessible.

#### 3.6.3 Process

The implementation processes observed across countries reflected different strategic logics, shaped by institutional design, risk appetite, and the maturity of genomics integration. Estonia followed a top-down, policy-aligned approach: national tendering, coordinated pilot design, and EHR workflow integration illustrated a streamlined innovation implementation pathway. Sweden adopted a pilot-first, scale-later model: RCCs and leading regions, like Uppsala, were empowered to test PRS in a controlled setting, with the expectation that successful models would inform national guidance. Spain displayed a multi-level process logic: while national inclusion of PRS in the Common Catalog remained unlikely in the short term, regions like Catalonia used scientific evidence (DECIDO) and clinical consensus to advance regional implementation. In Portugal, the dominant process was market-driven: private hospitals and labs moved ahead with PRS as part of bundled wellness services, while public actors remained procedurally constrained by INFARMED's lengthy HTA cycles. France followed a trial-to-policy pathway, relying on MyPeBS as a proof-of-concept, but lacked the policy agility to translate trial results into routine practice without formal HTA endorsement. Across cases, stakeholders emphasized that pilots, institutional learning cloops, and co-developed guidelines were crucial to overcoming initial resistance. Yet, process fragmentation, particularly between health policy and genomic research communities, remained a systemic barrier to coherent and scalable PRS implementation. Although Estonia is represented as the strongest implementation environment across all VIP dimensions in relative terms (Table 4), this does not indicate complete system readiness. Rather, it reflects comparative advantage within the study sample. In Estonia's case, centralized governance and highly integrated information systems create favorable conditions across value, information, and process dimensions; however, continued reliance on pilot-based evidence, evolving reimbursement arrangements, and the absence of fully routinized national screening pathways indicate that implementation remains in transition rather than complete.

**Table 4:** Summarizes relative cross-country strengths across the VIP dimensions. Being identified as the strongest implementation environment in a given dimension does not imply full readiness, but rather indicates comparative advantage relative to other countries in the sample.

VIP Dimension	Strongest Implementation Environment	Greatest Challenges
Value	Estonia, Sweden	Portugal, France
Information	Estonia	Spain, Portugal
Process	Estonia, Sweden (locally)	France, Spain

## 4 Discussion

This study explored the implementation of polygenic risk score (PRS)-based breast cancer screening across five European countries, revealing how institutional structures, information systems, and stakeholder dynamics shape the feasibility of genomic innovation in public health. By combining the Value–Information–Process (VIP) framework with selected dimensions of the Consolidated Framework for Implementation Research (CFIR), we provide a multilayered understanding of why certain health systems are better positioned than others to integrate PRS into routine screening workflows (23, 25, 33).

### 4.1 Linking frameworks: VIP and CFIR in action

The VIP framework allowed us to trace how stakeholders assign value, exchange and interpret information, and construct implementation pathways. CFIR, in turn, enriched this analysis by situating these elements within their outer (policy, economic) and inner (organizational, cultural) settings, while emphasizing the role of individual actors and change processes. For instance, while VIP helped us map the high value ascribed to PRS in Estonia and Sweden, CFIR clarified why implementation advanced in Estonia (strong leadership, digital readiness) but remained fragmented in Sweden (decentralized governance and slow guideline cycles).

Together, the two frameworks expose a critical insight: innovation adoption is not solely a function of scientific evidence or system capacity, but of strategic alignment across multiple levels of governance, practice, and belief. This reinforces Solaimani et al.'s (2017) contention that implementation success in networked enterprises requires co-evolution across actors, tools, and logics, not just top-down design.

### 4.2 Diverging logics of PRS adoption

Three broad models of PRS implementation emerge from our cross-case synthesis:

- Policy-Driven Integration (Estonia): Alignment between national genomics policy, biobank infrastructure, and digital referral logic enabled seamless PRS integration into screening processes with minimal clinical resistance.
- Pilot-to-Policy Translation (Sweden, Spain, France): Regions or national entities piloted PRS in controlled environments, awaiting sufficient real-world data and HTA signals to scale. This logic is slower but preserves institutional legitimacy.
- Market-Led Adoption (Portugal, parts of France): In the absence of public-sector agility, private actors led implementation through wellness and executive health packages. While agile, this raises concerns about equity, standardization, and long-term clinical utility.

These models reflect deeper institutional logics around innovation legitimacy, risk, and responsibility. Where some systems see PRS as a public good to be scaled through careful governance, others treat it as a consumer innovation that must prove its value in the market first.

### 4.3 Implementation challenges and facilitators

Across all five national cases, a consistent set of structural and operational challenges emerged in the implementation of PRS-based breast cancer screening (Table 5). Perhaps most universally cited was the absence of formal clinical guidelines for interpreting and acting on PRS results. Although risk scores were generally understood by genetic specialists, frontline physicians, particularly general practitioners and radiologists, expressed hesitation or discomfort in applying probabilistic risk data within routine screening decisions. This gap in clinical confidence, tied closely to professional training norms and legal responsibilities, served as a critical bottleneck in translating genomic insight into medical action.

**Table 5:** Key opportunities and challenges for implementing PRS-based breast cancer screening in each of the five BRIGHT countries.

Country	Key Opportunities	Key Challenges
Estonia	Centralized governance; strong digital health infrastructure; Biobank; prior PRS pilot and scientific success	Limited clinical capacity; lack of widespread genetic literacy among clinicians
Sweden	Regional innovation capacity; strong biobanks; patient advocacy support	Decentralized system limits national scaling; variation in readiness across regions
Portugal	Private sector agility; supportive civil society; centralized reimbursement system	Slow HTA processes; limited public reimbursement; low clinician familiarity with PRS
Spain	Active regional pilots (e.g., Catalonia); flexible local adoption pathways	Highly fragmented process; lack of centralized integration; limited genetics training
France	National genomic strategy; RIHN mechanism for early-stage funding; strong research ecosystem	Rigid regulatory pathways; slow HTA decisions; unclear integration into national screening

Another recurring obstacle was low genetic literacy among healthcare professionals. In most countries, there is no dedicated clinical genetics specialty, and genomic content is minimally covered in medical curricula. This created reliance on small numbers of experts, whose capacity was quickly exceeded in pilot settings. Relatedly, many systems lacked supportive infrastructure, such as automated decision-support tools or communication templates, that could aid in the dissemination and understanding of complex risk information.

Information system fragmentation also presented a significant barrier. While Estonia and Sweden had advanced digital infrastructure with PRS-compatible electronic health records, most other countries relied on screening registries or local hospital systems that lacked interoperability. Even in France, where sequencing capacity is world-class, integration between trial platforms (e.g., MyPeBS), hospital IT, and national data repositories remains limited. This disconnection hampers real-time data sharing and makes it difficult to embed PRS in the same clinical workflow as traditional mammography.

In addition, the health technology assessment (HTA) and reimbursement processes were widely described as slow, rigid, and disconnected from innovation cycles. The evidentiary standards for inclusion in public benefit packages were perceived by many interviewees as disproportionately high for preventive tools, especially those grounded in probabilistic rather than deterministic logic. Without robust cost-effectiveness evidence and formal institutional endorsement, PRS has so far remained in a policy limbo in many health systems. However, ongoing analyses from the BRIGHT project are expected to provide new evidence on the economic implications of PRS-based breast cancer screening and may contribute to strengthening the currently limited evidence base supporting its clinical and policy adoption. Yet, despite these challenges, several facilitators supported partial or early-stage implementation. Countries with strong institutional coordination between genomics research, digital health, and public health agencies, like Estonia and parts of Sweden, were able to transition more decisively from pilot to practice. Regional autonomy, particularly in Spain and Sweden, allowed for localized innovation even in the absence of national consensus. Civil society organizations, such as EVITA in Portugal and Unicancer in France, have also played an important role in increasing patient awareness, shaping public narratives, and legitimizing personalized prevention. Where public investment in digital infrastructure and biobanking was already in place, stakeholders reported higher confidence in both the technical feasibility and the governance capacity of PRS integration.

### 4.4 Implementation theory and strategic recommendations

This study contributes to the growing field of implementation science by demonstrating how structured analytical frameworks can illuminate the challenges of translating genomic innovation into diverse healthcare contexts. Specifically, it shows that the Value–Information–Process (VIP) model, originally developed to study business model dynamics in networked enterprises, can be effectively adapted to public health genomics (23–25). When layered with insights from the Consolidated Framework for Implementation Research (CFIR), the VIP model gains additional depth, particularly in articulating how outer and inner contextual factors, such as regulatory environments, organizational capacity, and actor engagement, shape implementation trajectories.

Importantly, the study moves beyond descriptive country comparisons to identify recurring patterns in the logic of PRS adoption. It suggests that the presence of sequencing capacity, political will, or even strong pilot data is not sufficient. What matters more is the alignment between stakeholder value narratives, interoperable information infrastructures, and coherent process pathways. In systems where these three dimensions are synchronized, such as Estonia, implementation proceeds relatively easily. Where misalignment persists, such as in France or Portugal, adoption is more fragmented, opportunistic, or delayed.

By empirically grounding this analysis in over ninety interviews across five distinct health systems, the study offers a practical typology of PRS implementation pathways. It also highlights polygenic risk-based screening as a proxy for deeper tensions within public health innovation: between prevention and treatment, personalization and equity (36, 37), experimentation and regulation.

For policymakers, these findings underscore the importance of dual-track strategies that pair controlled, evidence-generating pilots with proactive policy mechanisms, such as conditional reimbursement or adaptive HTA (35), to reduce the lag between innovation and access. For researchers, the study suggests a need to further investigate how genomics fits within evolving definitions of public value, especially as risk-based models expand into other disease areas.

Ultimately, this research positions PRS-based screening not just as a technical intervention, but as a test case for how health systems respond to the promises and pressures of personalized medicine. It offers both a lens and a roadmap for navigating that terrain.

### 4.5 Trustworthiness and limitations

Several considerations should be taken into account when interpreting the findings. Stakeholder representation differed across countries, reflecting differences in access, national implementation contexts, and the roles of actors involved in PRS-based screening. As a result, some stakeholder groups, such as regulatory authorities or notified bodies, were not represented in all country samples, including Portugal and Estonia. While the available interviews provided rich insights into implementation conditions, these differences may have shaped the level of detail available for specific aspects, particularly regulatory and reimbursement processes. France was included as an analytically derived case, drawing on documentary material, published evidence, and relevant policy and pilot initiatives rather than primary interview data. This approach allowed France to be included as an important comparator case, although the findings should be interpreted in light of this data source difference. To enhance trustworthiness, interview findings were triangulated with policy documents, pilot evidence, and research team discussions, and preliminary interpretations were validated through member-checking with selected key stakeholders. These considerations do not undermine the comparative value of the study but indicate where future research could deepen country-specific implementation evidence.

### 4.6 Conclusion and future research

Our findings suggest that successful PRS implementation is not primarily a function of sequencing capacity or clinical innovation, but rather of strategic alignment across multiple layers of the healthcare ecosystem. In countries such as Estonia, and potentially Sweden, favorable conditions for PRS adoption are supported by strong alignment between national genomics policy, digital infrastructure, and stakeholder engagement, although implementation remains at the pilot or preparatory stage in Sweden. In contrast, systems characterized by bureaucratic inertia (France), procedural fragmentation (Spain), or public–private dualism (Portugal) face greater barriers in translating pilot initiatives into routine practice, even when clinical interest is strong. Three core insights emerge from this cross-case analysis. First, value narratives matter: how stakeholders define the purpose and utility of PRS, whether in terms of equity, efficiency, or innovation, shapes the pace and direction of implementation. Second, data readiness is essential: without interoperable electronic health records, integrated biobanks, and accessible interpretation tools, even promising genomic innovations are unlikely to scale within routine healthcare. Third, process design is inherently institutional and political: national HTA procedures, pilot funding mechanisms, and the presence of clinical guidelines all influence whether PRS becomes integrated into cancer prevention pathways or remains a niche innovation. At a broader level, PRS-based screening represents more than a technical intervention. It offers a lens into how health systems manage the evolving intersection of genomics, prevention, and personalized medicine. By enabling the reuse of genomic data for additional personalized prevention services, it also lays the groundwork for broader secondary uses of health data within population health strategies. In this sense, PRS functions both as a test of institutional agility and coordination, and as an opportunity to expand the scope of preventive care in the twenty-first century.

## 5 Future research agenda

While this study offers a structured, multi-country analysis, several areas warrant further investigation. First, future work should track the long-term outcomes of PRS pilots, both in terms of clinical effectiveness (e.g., stage at diagnosis) and system-level impacts (e.g., cost-effectiveness, budget impact, screening adherence, psychological well-being). These metrics will be crucial to securing HTA approval and public reimbursement.

Second, additional research is needed on risk communication and public engagement, particularly in relation to probabilistic information and its effects on decision-making across demographic groups. Understanding how women interpret and act on PRS data will be essential to designing equitable screening pathways.

Third, there is a need to explore the transferability of PRS-based screening models to other disease areas, such as other cancers, cardiovascular disease or diabetes (34, 38, 39), where risk stratification could similarly inform preventive strategies. Doing so will help determine whether PRS represents a domain-specific innovation or a more generalizable paradigm for risk-based health planning that is likely to become even more cost-effective with each added service.

Finally, future studies should focus on cross-sector implementation partnerships, including insurers, employers, and digital health firms, and how these actors might support or complicate the delivery of genomic prevention services. As public systems grapple with capacity constraints, such hybrid models may become increasingly relevant.

## Data Availability

The original contributions presented in the study are included in the article/Supplementary Material, further inquiries can be directed to the corresponding author.
